# Quantification of Aortic Valve Fibrotic and Calcific Tissue from CTA: Prospective Comparison with Histology

**DOI:** 10.1148/radiol.240229

**Published:** 2024-08-13

**Authors:** Kajetan Grodecki, Anna Olasińska-Wiśniewska, Agata Cyran, Tomasz Urbanowicz, Jacek Kwieciński, Jolien Geers, Balaji K. Tamarappoo, Bartłomiej Perek, Radosław Gocoł, Joanna Nawara-Skipirzepa, Marek Jemielity, Janusz Kochman, Wojciech Wojakowski, Barbara Górnicka, Piotr J. Slomka, Hasan Jilaihawi, Raj R. Makkar, Zenon Huczek, Damini Dey

**Affiliations:** From the First Department of Cardiology (K.G., J. Kochman, Z.H.) and Department of Pathology (A.C., B.G.), Medical University of Warsaw, Warsaw, Poland; Departments of Biomedical Sciences and Medicine, Biomedical Imaging Research Institute, Cedars-Sinai Medical Center, 116 N Robertson Blvd, Suite 400, Los Angeles, CA 90048 (K.G., J.G., P.J.S., D.D.); Department of Cardiac Surgery and Transplantology, Poznan University of Medical Sciences, Poznan, Poland (A.O.W., T.U., B.P., M.J.); Department of Interventional Cardiology and Angiology, Institute of Cardiology, Warsaw, Poland (J. Kwieciński); Department of Cardiology, Centrum voor Hart- en Vaatziekten, Universitair Ziekenhuis Brussel, Vrije Universiteit Brussel, Brussels, Belgium (J.G.); Department of Cardiology, Banner University Medical Center, Indianapolis, Ind (B.K.T.); Department of Cardiac Surgery (R.G.) and Division of Cardiology and Structural Heart Diseases (J.N.S., W.W.), Medical University of Silesia, Katowice, Poland; and Smidt Heart Institute, Cedars-Sinai Medical Center, Los Angeles, Calif (H.J., R.R.M.).

## Abstract

**Background:**

Quantifying the fibrotic and calcific composition of the aortic valve at CT angiography (CTA) can be useful for assessing disease severity and outcomes of patients with aortic stenosis (AS); however, it has not yet been validated against quantitative histologic findings.

**Purpose:**

To compare quantification of aortic valve fibrotic and calcific tissue composition at CTA versus histologic examination.

**Materials and Methods:**

This prospective study included patients who underwent CTA before either surgical aortic valve replacement for AS or orthotopic heart transplant (controls) at two centers between January 2022 and April 2023. At CTA, fibrotic and calcific tissue composition were quantified using automated Gaussian mixture modeling applied to the density of aortic valve tissue components, calculated as [(volume/total tissue volume) × 100]. For histologic evaluation, explanted valve cusps were stained with Movat pentachrome as well as hematoxylin and eosin. For each cusp, three 5-µm slices were obtained. Fibrotic and calcific tissue composition were quantified using a validated artificial intelligence tool and averaged across the aortic valve. Correlations were assessed using the Spearman rank correlation coefficient. Intermodality and interobserver variability were measured using the intraclass correlation coefficient (ICC) and Bland-Altman plots.

**Results:**

Twenty-nine participants (mean age, 63 years ± 10 [SD]; 23 male) were evaluated: 19 with severe AS, five with moderate AS, and five controls. Fibrocalcific tissue composition strongly correlated with histologic findings (*r* = 0.92; *P* < .001). The agreement between CTA and histologic findings for fibrocalcific tissue quantification was excellent (ICC, 0.94; *P* = .001), with underestimation of fibrotic composition at CTA (bias, −4.9%; 95% limits of agreement [LoA]: −18.5%, 8.7%). Finally, there was excellent interobserver repeatability for fibrotic (ICC, 0.99) and calcific (ICC, 0.99) aortic valve tissue volume measurements, with no evidence of a difference in measurements between readers (bias, −0.04 cm^3^ [95% LoA: −0.27 cm^3^, 0.19 cm^3^] and 0.02 cm^3^ [95% LoA: −0.14 cm^3^, 0.19 cm^3^], respectively).

**Conclusion:**

In a direct comparison, standardized quantitative aortic valve tissue characterization at CTA showed excellent concordance with histologic findings and demonstrated interobserver reproducibility.

Clinical trial registration no. NCT06136689

© The Author(s) 2024. Published by the Radiological Society of North America under a CC BY 4.0 license.

*Supplemental material is available for this article.*

See also the editorial by Almeida in this issue.

## Graphical abstract

**Figure:**
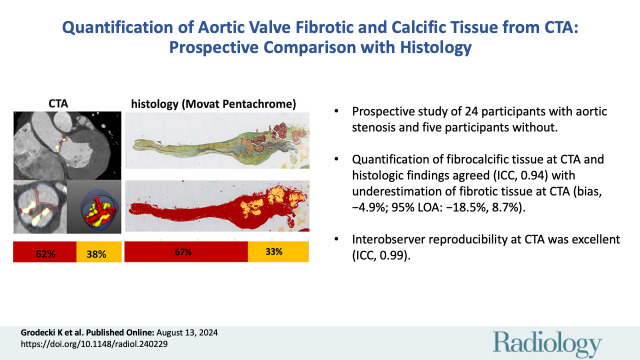

**Summary**

In participants with aortic stenosis, semiautomated quantification of aortic valve fibrotic and calcific tissue composition at CT angiography showed excellent concordance with histologic findings and demonstrated interobserver reproducibility.

**Key Results**

■ In this prospective study of 24 participants with aortic stenosis and five participants without stenosis, quantification of aortic valve fibrocalcific tissue at CT angiography (CTA) and histologic findings showed excellent agreement (intraclass correlation coefficient [ICC], 0.94; *P* = .001), with underestimation of fibrotic tissue composition at CTA (bias, −4.9%; 95% limits of agreement: −18.5%, 8.7%).
■ The interobserver reproducibility for measuring fibrotic and calcific tissue volumes at CTA was excellent (ICC, 0.99 and 0.99, respectively).

## Introduction

Aortic stenosis (AS) is the most common valvular disease in developed countries (1,2). Increased fibrosis and the accumulation of calcium contribute to valvular thickening and alter the biomechanical properties of the aortic valve complex, leading to progressive valve narrowing and dysfunction (3,4). Recent studies have demonstrated that aortic valve calcium quantified at electrocardiogram-gated noncontrast cardiac CT is a robust tool for assessing AS severity in individuals in whom echocardiography is inconclusive and that the burden of valvular calcium can be used to predict both disease progression and clinical events (5–7). Although increased volume of aortic valve fibrosis is a common precursor to stenosis and has been associated with increased cardiovascular mortality, its utility as an imaging marker has not yet been established (8,9).

A previous study (10) showed that fibrotic and calcific characteristics of aortic valve thickening measured manually at CT angiography (CTA) correlated with peak aortic jet velocity on echocardiograms in patients with AS. Further, in a multicenter study, manual quantification of aortic valve composition was found to distinguish between classic high-gradient and low-flow low-gradient AS and predict postprocedural outcomes following transcatheter aortic valve replacement (11). Quantitative fibrotic and calcified aortic valve tissue measurements have yet to be validated against quantitative histologic findings, precluding their use in clinical practice or research trials. Therefore, the aim of this study was to compare the performance of a semiautomated imaging algorithm for complete aortic valve tissue characterization with quantitative histologic findings as the reference standard.

## Materials and Methods

The Role of Aortic Valve Composition in Pathophysiology and Diagnosis of Aortic Stenosis study, or COMP-AS (ClinicalTrials.gov registration no. NCT06136689), is a secondary analysis of a prospective study conducted in accordance with the Declaration of Helsinki and approved by the local ethics committee (Bioethics Committee of Medical University of Warsaw, KB/126/2021). All participants provided written informed consent.

### Study Participants

**Figure 1:**
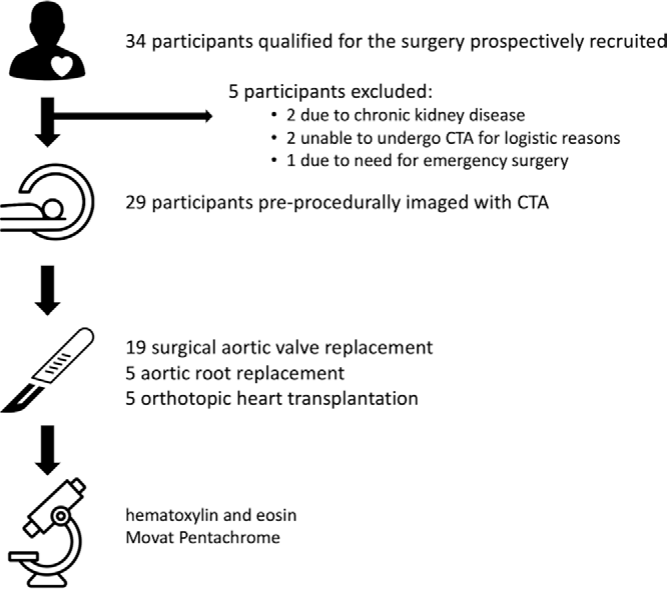
Study flowchart. CTA = CT angiography.

This study included consecutive patients who underwent CTA imaging for presurgical assessment before aortic valve replacement or heart transplant at two university teaching hospitals between January 2022 and April 2023. Patients undergoing surgical aortic valve replacement for different phenotypes of severe AS (high-flow high-gradient, low-flow low-gradient with low left ventricle ejection fraction, and low-flow low-gradient with preserved left ventricle ejection fraction) or those with moderate AS with coexisting aortic aneurysm as part of the Bentall procedure were eligible for inclusion (Fig 1). Definition of terms is presented in Appendix S1. Patients were excluded if they were unable to give informed consent, had impaired renal function (estimated glomerular filtration rate <30 mL/min/1.73 m^2^), or had known rheumatic heart disease.

Participants who underwent orthotopic heart transplants and were determined to be nonstenotic based on historical echocardiography reports were included as controls. Aortic valves were excised from all participants during surgery and stored at a biobank for further staining at the histopathology laboratory.

Fibrotic and calcific components assessed at histology in the aortic valve were compared with corresponding CTA-derived measurements to determine agreement between the two methods.

### CTA Protocols

All participants underwent contrast-enhanced cardiac CTA for presurgical screening within 30 days before surgery. Details of the scan protocol are presented in Appendix S1.

### Image Analysis

Quantitative analysis of aortic valve composition was based on presurgical planning CTA images using semiautomated software (Autoplaque, version 2.51; Cedars-Sinai Medical Center) and a standard mediastinal window (width, 400 HU; level, 40 HU).

The version of Autoplaque software used in this study incorporates a probabilistic model that allows for automatic scan-specific thresholding and identification of tissues with different voxel intensity within the aortic valve region of interest. Details of scan-specific modeling are presented in Appendix S1. Fibrocalcific volume was the sum of calcific and fibrotic tissue volumes. Volumes of respective tissue volumes are expressed in cubic centimeters. Tissue composition is expressed as a percentage and was calculated as [(tissue component volume/total tissue volume) × 100]. The fibrocalcific ratio was calculated by dividing the fibrotic tissue volume by the calcific tissue volume.

All tomographic analyses were performed at the Cedars-Sinai Medical Center core laboratory by two readers (K.G. and J.G., with 7 and 5 years of experience in cardiovascular CT) blinded to histologic and clinical data. Both readers assessed the whole data set independently.

### Histologic Assessment

**Figure 2:**
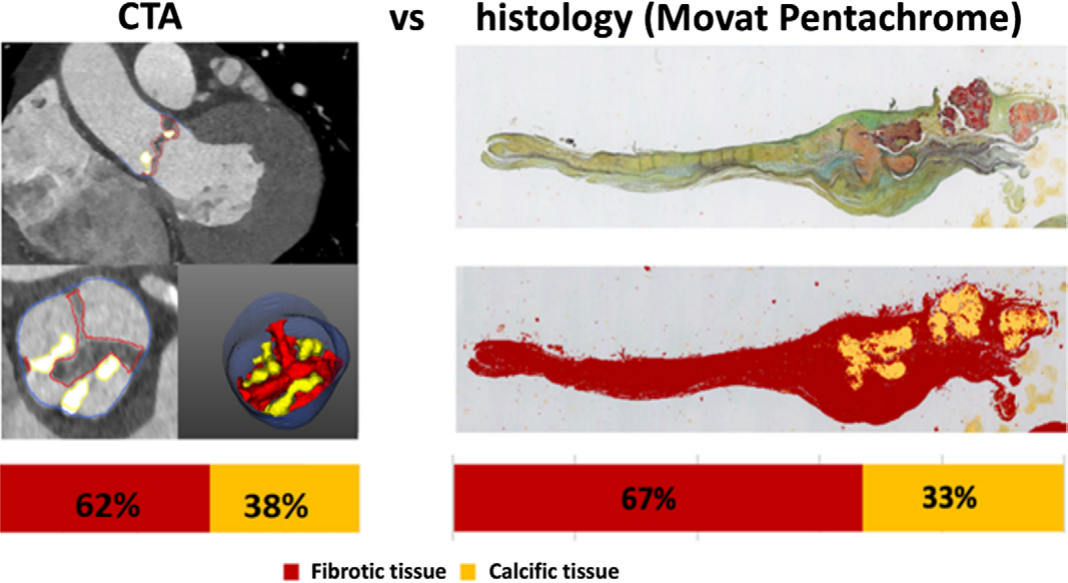
Example images show comparison of tissue quantification of the aortic valve from CT angiography (CTA) and histologic assessment with use of Movat pentachrome staining in a 75-year-old male participant with aortic stenosis. For CTA, a region of interest was defined between the lower coronary ostium and the virtual basal ring using coronal and axial planes; scan-specific thresholds were used for fibrotic (red) and calcific (yellow) tissues. For quantitative histologic assessment, every valve was evaluated using nine nonconsecutive slices (three per cusp), and the proportion of fibrotic (red) and calcific (yellow) tissues was averaged across the whole set. The bottom right CTA image is not supported by artificial intelligence.

Histologic preparation and analysis were performed at the Department of Pathology, Medical University of Warsaw, by a certified pathologist (A.C.) with more than 15 years of experience who was blinded to the CTA measurements. Nine slices per valve were stained with Movat pentachrome as well as hematoxylin and eosin (12). All slices were stained in a single batch to decrease the sample variability. Slices stained with Movat pentachrome were quantitatively evaluated using an artificial neural network for pixel classification (Fig 2). Semiquantitative assessment of tissues stained with Movat pentachrome and hematoxylin and eosin was also performed using Warren-Yong scores (13). Details of the histologic protocol and artificial intelligence–enabled quantification of slices are presented in Appendix S1.

### Statistical Analysis

Participant characteristics representing categorical variables are presented as absolute numbers with percentages and were compared between groups of participants with severe AS, moderate AS, and heart transplant with use of the Fisher exact test. Data were tested for normality using the Shapiro-Wilk test. Continuous data are expressed as means ± SDs or medians with IQRs depending on the distribution and, for participant characteristics, were compared between participants with severe AS, moderate AS, and heart transplant with use of one-way analysis of variance or the Kruskal-Wallis test, as appropriate. The Wilcoxon rank sum test was used for paired sample analysis comparing tissue composition between CTA and histologic assessment. Aortic valve tissue composition was compared between participants with severe AS, moderate AS, and heart transplant and across respective Warren-Yong scores with use of the Kruskal-Wallis test with Bonferroni correction for post hoc comparisons between two specific groups. Correlations between continuous variables were assessed using the Spearman rank correlation coefficient, with values less than 0.5 indicative of poor correlation; values between 0.5 and 0.74, moderate correlation; between 0.75 and 0.9, good correlation; and greater than 0.90, excellent correlation. The correlation between composition and echocardiographic parameters was calculated excluding participants with heart transplant. Intermodality and interobserver agreement were measured using an intraclass correlation coefficient (ICC), with values less than 0.5 indicative of poor agreement; values between 0.5 and 0.74, moderate agreement; between 0.75 and 0.9, good agreement; and greater than 0.90, excellent agreement. Intermodality and interobserver variability were measured using Bland-Altman plots with mean bias and limits of agreement (LoA). All probability values were two-tailed, and *P* < .05 was considered indicative of statistically significant difference. Data were processed using the SPSS software (version 25, IBM) and MedCalc (version 22, MedCalc Software).

## Results

### Study Participants

A total of 34 participants qualified for surgical aortic valve replacement or heart transplant were enrolled. Among those participants, five were excluded because they were unable to undergo preprocedural CTA (two due to chronic kidney disease, two due to logistic reasons, and one due to the need for emergency surgery). Finally, 29 participants (mean age, 63 years ± 10 [SD]; 23 male, six female) who underwent CTA at a median of 23 days (IQR, 15–30 days) ahead of the surgery were included in the study (Fig 1). Of the 29 participants, 19 (66%) had severe AS and were treated with surgical aortic valve replacement (15 with high-gradient, two with paradoxical low-flow low-gradient, and two with classic low-flow low-gradient), five (17%) had moderate AS and underwent aortic root replacement (Bentall procedure), and five (17%) were heart transplant recipients with no stenosis who were included in the control group (two for dilated cardiomyopathy, two for ischemic heart failure, and one for restrictive cardiomyopathy). Clinical characteristics are listed in Table 1.

**Table 1:**
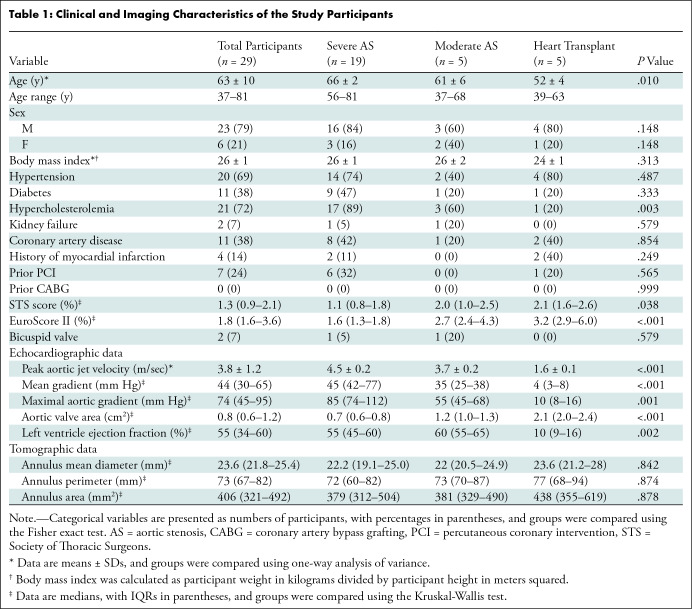
Clinical and Imaging Characteristics of the Study Participants

### Tomographic Tissue Characterization of Stenotic Valves

Fibrocalcific tissue volume differed between participants with severe AS, moderate AS, and heart transplant (median, 1.86 cm^3^ [IQR, 1.32–3.14 cm^3^] vs 1.47 cm^3^ [IQR, 0.95–2.42 cm^3^] vs 0.76 cm^3^ [IQR, 0.52–0.95 cm^3^], respectively; *P* = .009) (Fig S4). The difference between participants with severe AS, moderate AS, and heart transplant was noted also for fibrotic (median, 1.18 cm^3^ [IQR, 0.91–1.80 cm^3^] vs 1.36 cm^3^ [IQR, 0.89–2.16 cm^3^] vs 0.71 cm^3^ [IQR, 0.51–0.88 cm^3^]; *P* = .021) and calcific (median, 0.79 cm^3^ [IQR, 0.22–1.01 cm^3^] vs 0.11 cm^3^ [IQR, 0.04–0.29 cm^3^] vs 0.04 cm^3^ [IQR, 0.01–0.07 cm^3^]; *P* = .001) tissue volumes (Fig S4).

### Comparison of Tissue Composition Quantified from CTA and Histologic Assessment

**Figure 3:**
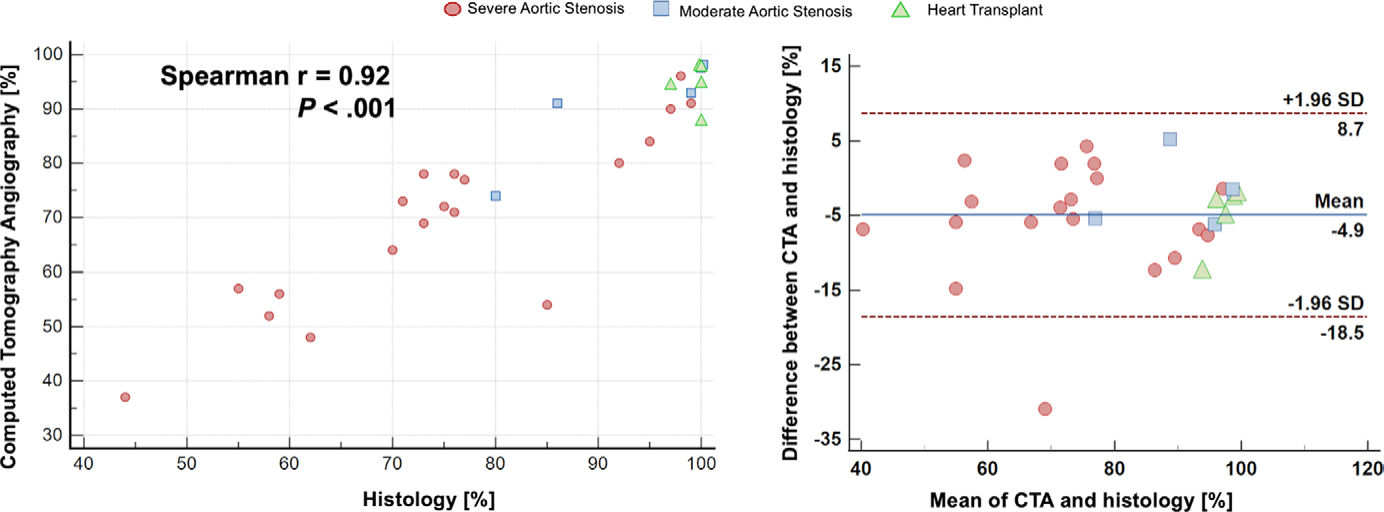
Scatterplot (left) and Bland-Altman plot (right) compare the quantification of fibrotic tissue composition of the aortic valve at CT angiography (CTA) and quantitative histologic findings. The Spearman rank correlation coefficient shows a strong correlation between CTA and quantitative histology (*r* = 0.92; *P* < .001). The Bland-Altman plot shows underestimation of fibrotic tissue composition measurement at CTA compared with quantitative histologic assessment (bias, −4.9%; 95% limits of agreement: −18.5%, 8.7%). Calcific tissue composition (percentage) is determined by subtracting the fibrotic tissue composition from 100%; the corresponding Bland-Altman plot is presented in Figure S7.

The proportion of the aortic valve that was comprised of fibrotic tissue was lower at CTA (median, 77% [IQR, 66%–93%]) than histology in the overall study sample (median, 84% [IQR, 72%–98%]; *P* < .001). Furthermore, the proportion of fibrotic tissue was higher at CTA than histology in participants with severe AS (median, 75% [IQR, 62%–92%] vs 72% [IQR, 56%–80%]; *P* = .003) and heart transplant (median, 100% [IQR, 99%–100%] vs 95% [IQR, 91%–98%]; *P* = .043), but not moderate AS (median, 99% [IQR, 83%–100%] vs 93% [IQR, 83%–98%]; *P* = .225) (Fig S5). The correlation between CTA and histologic assessment for fibrotic tissue quantification was excellent (*r* = 0.92; *P* < .001) (Fig 3). Agreement between CTA and histologic assessment was also excellent, with an ICC of 0.94 (95% CI: 0.77, 0.99; *P* = .001). Bland-Altman analysis demonstrated a coefficient of repeatability of 16.5 and a bias of −4.9% (95% LoA: −18.5%, 8.7%) between CTA and quantitative histologic assessment (Fig 3).

**Figure 4:**
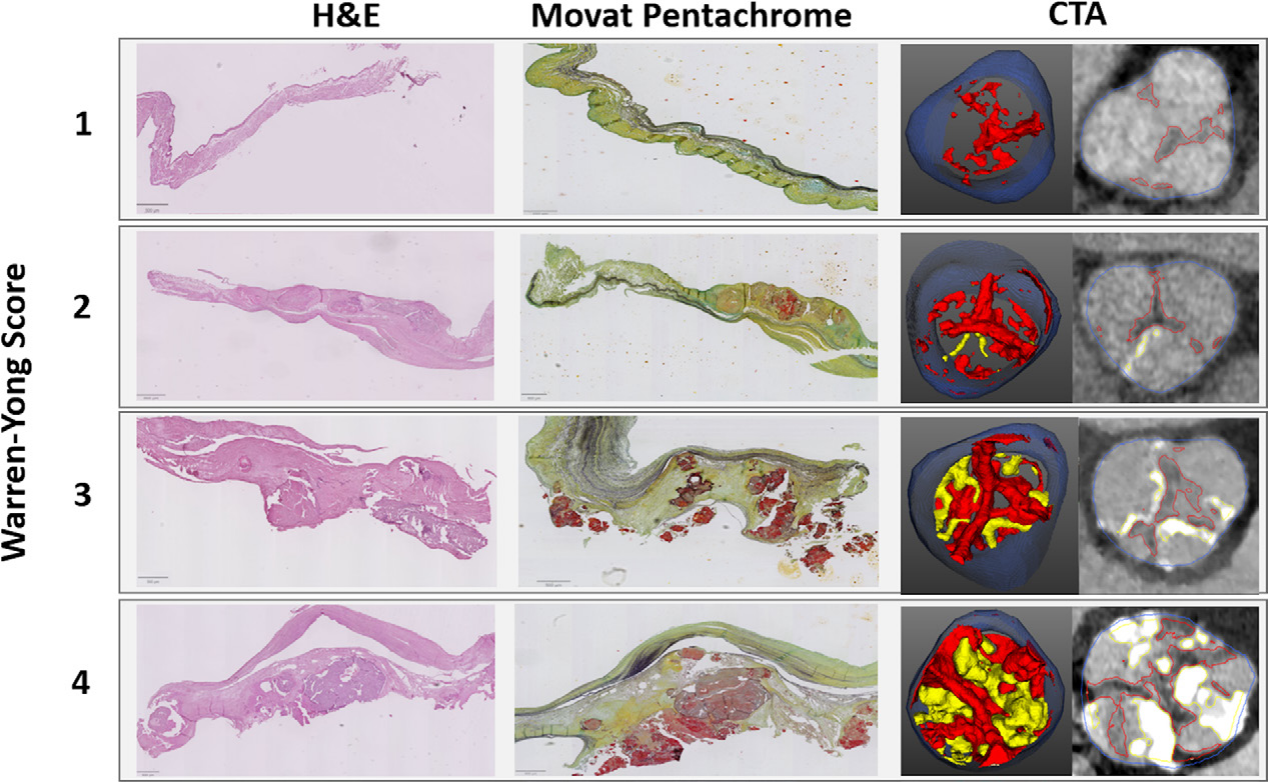
Representative examples of Warren-Yong scores in hematoxylin and eosin (H&E) staining, Movat pentachrome staining, and CT angiography (CTA). On slides stained with hematoxylin and eosin and Movat pentachrome, calcification of aortic cusps was semiquantitatively assessed (1 = absence of calcification; 2 = mild valve thickening and early nodular calcification; 3 = moderate thickening with many calcified nodules; and 4 = severe thickening with many calcified nodules). On CTA images, fibrotic tissue is shown in red, and calcific tissue is shown in yellow. CTA images in the left column are not supported by artificial intelligence.

**Figure 5:**
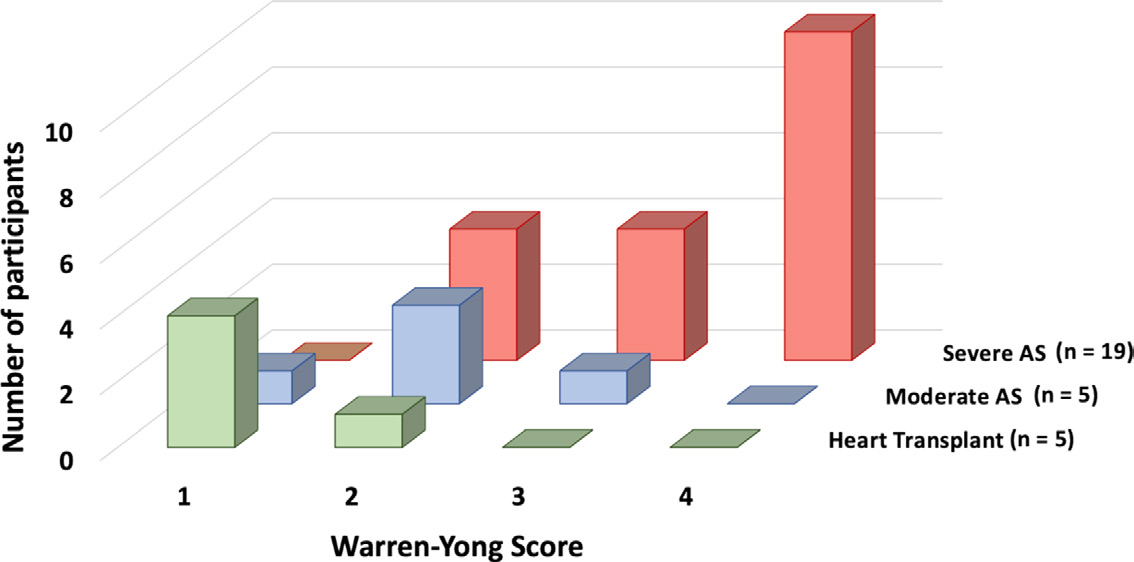
Bar graph shows the number of participants with severe aortic stenosis (AS), moderate AS, and heart transplant in the respective Warren-Yong scores.

In a semiquantitative histologic evaluation of excised aortic valve samples using Warren-Yong scores (Fig 4), the majority of participants with severe AS (52% [10 of 19]) received the highest grade (grade 4); the majority of participants with moderate AS (60% [three of five]) were assigned a moderate grade (grade 2), and the majority of participants who received heart transplants (80% [four of five]) were allocated to the lowest grade (grade 1) (Fig 5). It was observed that higher Warren-Yong scores corresponded to higher fibrocalcific, fibrotic, and calcific tissue volumes measured at quantitative histologic assessment, and differences in median volumes stratified by Warren-Yong scores for each are shown in Figure S6.

### Correlation Between Aortic Valve Composition Parameters and Aortic Valve Weight

No evidence of a difference in the weight of the aortic valve was observed between the participants with severe AS, moderate AS, and heart transplant (mean, 2.35 g ± 0.56 vs 2.66 g ± 0.49 vs 2.08 g ± 0.46; *P* = .467). There was a moderate correlation between valvular weight and CTA-derived calcific tissue volume (Spearman *r* = 0.61; *P* = .001) as well as a CTA-derived fibrocalcific volume (Spearman *r* = 0.66; *P* < .001).

### Association With Echocardiographic Parameters

CTA-derived fibrocalcific volume demonstrated a moderate correlation with mean transvalvular gradient (Spearman *r* = 0.75; *P* < .001), peak transvalvular velocity (Spearman *r* = 0.65; *P* < .001), and aortic valve area (Spearman *r* = −0.58; *P* = .003) in participants with severe and moderate AS (Table 2). Similarly, moderate correlations were observed between calcific tissue quantified from histologic assessment and mean transvalvular gradient (Spearman *r* = 0.70; *P* < .001), peak transvalvular velocity (Spearman *r* = 0.66; *P* < .001), and aortic valve area (Spearman *r* = −0.54; *P* = .006) in participants with severe and moderate AS.

**Table 2:**
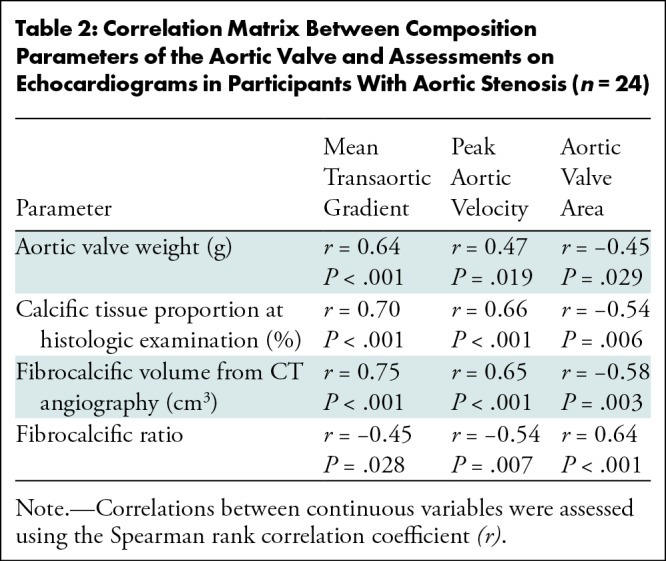
Correlation Matrix Between Composition Parameters of the Aortic Valve and Assessments on Echocardiograms in Participants With Aortic Stenosis (*n* = 24)

### Reproducibility of Aortic Valve Composition Assessment from CTA

**Figure 6:**
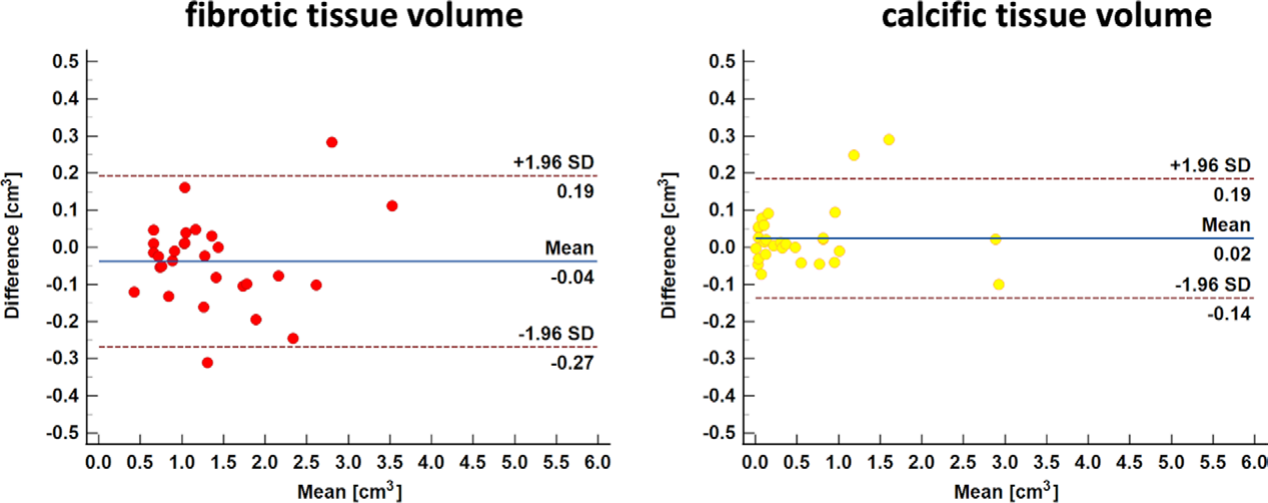
Bland-Altman plots of interobserver repeatability between two readers for assessing fibrotic and calcific tissue volume measurements at CT angiography. No evidence of a difference in measurements was observed between the two readers for either fibrotic (bias, −0.04 cm^3^; 95% limits of agreement [LoA]: −0.27 cm^3^, 0.19 cm^3^) or calcific (bias, 0.02 cm^3^; 95% LoA: −0.14 cm^3^, 0.19 cm^3^) tissue volumes.

There was excellent interobserver repeatability for fibrotic tissue volume measurement, with an ICC of 0.99 (95% CI: 0.97, 0.99; *P* < .001). Additionally, Bland-Altman analysis showed that the two readers achieved a coefficient of repeatability of 0.23 and mean bias of −0.04 cm^3^ (95% LoA: −0.27 cm^3^, 0.19 cm^3^) for assessment of fibrotic tissue volume (Fig 6). Interobserver repeatability for calcific tissue volume was also excellent, with an ICC of 0.99 (95% CI: 0.99, 0.99; *P* < .001), and Bland-Altman analysis showed that the two readers achieved a coefficient of repeatability of 0.16 and mean bias of −0.02 cm^3^ (95% LoA: −0.14 cm^3^, 0.19 cm^3^) (Fig 6).

## Discussion

Quantifying the fibrotic and calcific composition of the aortic valve at CT angiography (CTA) can be useful for assessing disease severity and outcomes of patients with aortic stenosis; however, it has not yet been validated against quantitative histologic findings. The aim of this prospective study was to compare quantitative aortic valve tissue characterization at CTA versus histology. We found that *(a)* quantification of aortic valve fibrocalcific tissue at CTA and histology showed excellent agreement (intraclass correlation coefficient [ICC], 0.94; *P* = .001), with underestimation of fibrotic composition at CTA (bias, −4.9%; 95% limits of agreement: −18.5%, 8.7%), and *(b)* the interobserver reproducibility for measuring fibrotic and calcific tissue volumes at CTA were excellent (ICC, 0.99 and 0.99, respectively).

CTA has recently emerged as a tool for quantifying the calcific and fibrotic tissue components of the aortic valve (14,15). Indeed, we show that severe AS is associated with higher volumes of both fibrotic (median, 1.18 cm^3^ vs 0.71 cm^3^; *P* = .019) and calcific (median, 0.79 cm^3^ vs 0.04 cm^3^; *P* = .001) tissues as compared with participants with heart transplant. Interestingly, we found no evidence of a difference in fibrotic tissue volume between participants with severe AS and those with moderate AS (median, 1.18 cm^3^ [IQR, 0.91–1.80 cm^3^] vs 1.36 cm^3^ [IQR, 0.89–2.16 cm^3^]; *P* = .999). While it may be an incidental finding due to the small sample size, these results reflect a study on the molecular pathophysiology of the disease (16). It is suggested that increased fibrosis promotes calcification, but in the more advanced stages of the disease, while calcific volume increases rapidly, the fibrotic volume remains stable. Alternatively, our observation may be explained by photon starvation artifacts at CTA caused by the high-density calcium deposits in participants with severe stenosis, resulting in adjacent areas with attenuation below the detection threshold for fibrotic tissue.

Previous studies have found that measurements of tissue composition from CTA improve differentiation between the types of AS and can also help detect disparities in bi- and tricuspid valves, but these studies lacked validation against histologic findings (11,17). One study by Cartlidge et al (10) compared tissue measurements at CTA with histologic findings and found aortic valve fibrosis to spatially correlate with areas of noncalcific leaflet thickening at CT, but only visual assessment was used rather than more objective quantitative methods to evaluate stained slices. Visual scoring is inherently burdened with limited accuracy and reproducibility as compared with the artificial intelligence–enabled quantitative approach (18) used in our study. Moreover, the authors evaluated only a single aortic cusp in each participant, consequently introducing a potential bias, as the disease is rarely uniform in distribution across all three cusps (10). Finally, we used an automated approach for quantifying fibrocalcific volume with adaptive thresholds for respective tissue volumes. This decreased the time required from 45 minutes in the study requiring manual segmentation by Cartlidge et al (10) to 5 minutes in our study.

The important role of fibrotic tissue in AS pathophysiology was also evidenced through quantitative analysis of histologic samples in a noncontrast CT study focusing on aortic valve calcium scores (16). We demonstrate the close agreement between CTA and quantitative histologic measurements assisted with artificial intelligence in participants at different stages of the disease. Notably, Bland-Altman analysis of CTA versus quantitative histologic findings showed that CTA appears to underestimate the proportion of the fibrotic tissue within the aortic valve (mean bias of −4.9%). Such difference may result from volumetric measurement of calcific tissue at CTA, which affects the proportion of the respective components and reflects the natural difference between three-dimensional in vivo imaging with CTA and the histologic analysis.

Our results showed a comparable correlation of aortic valve weight with both the fibrocalcific and calcific volumes from CTA. However, this could be affected by the preponderance of male participants in our study. Weighing of the aortic valve has been used as a surrogate marker to account for variability in calcium detection in single histologic slices (19). This method has been used widely in studies on aortic valve calcium score and allowed the discovery of sex and valve morphologic characteristics as determinants of the weight of explanted valves (20). Despite similar transvalvular gradients in severe AS observed in both sexes, the lower valvular weight and calcific tissue contribution in women further supports the vital role of fibrotic tissue in the pathophysiology of AS (21). The predominance of valvular fibrosis in women was later demonstrated using a combination of noncontrast CT and quantification of dense connective tissue (16).

Our study has several limitations. First, our sample size was relatively small, especially for participants with moderate AS (*n* = 5) and controls (*n* = 5), and thus may have been underpowered for certain analyses. Second, women were underrepresented in our study (21% [six of 29 participants]), limiting the generalizability of the presented findings. Finally, aortic valve calcium score from noncontrast CT was unavailable for most participants; therefore, a direct comparison between aortic valve calcium scoring and CTA-derived fibrocalcific volume was not possible.

In conclusion, in this direct comparison, standardized quantitative aortic valve tissue characterization at CT angiography (CTA) showed excellent concordance with histologic findings and demonstrated interobserver reproducibility. Quantifying fibrocalcific tissue at CTA could aid in determining optimal timing for intervention; however, our findings require further validation in a multicenter study combining different stages of disease that would facilitate determining sex-specific thresholds for severe aortic stenosis.
